# Physical Activity, Step Counts, and Grip Strength in the Chinese Children and Families Cohort Study

**DOI:** 10.3390/ijerph17176202

**Published:** 2020-08-26

**Authors:** David Berrigan, Ailing Liu, Britni R. Belcher, Ann Chao, Liwen Fang, Charles E. Matthews, Baohua Wang, Linhong Wang, Ning Wang, Yu Wang, Lichen Yang, Martha S. Linet, Nancy Potischman

**Affiliations:** 1 Division of Cancer Control and Population Sciences, National Cancer Institute, Rockville, MD 20850, USA; 2 National Institute for Nutrition and Health, Chinese Center for Disease Control and Prevention, Beijing 100050, China; liual@ninh.chinacdc.cn (A.L.); yanglichen28@126.com (L.Y.); 3 Department of Preventive Medicine, Keck School of Medicine, University of Southern California, Los Angeles, CA 90033, USA; bbelcher@usc.edu; 4 Center for Global Health, National Cancer Institute, National Institutes of Health, Bethesda, MD 20850, USA; ann.chao@nih.gov; 5 National Center for Chronic and Noncommunicable Disease Control and Prevention, Chinese Center for Disease Control and Prevention, Beijing 100050, China; fangliwen@ncncd.chinacdc.cn (L.F.); baohua2000@126.com (B.W.); linhong@chinawch.org.cn (L.W.); wangning@ncncd.chinacdc.cn (N.W.); 6 Division of Cancer Epidemiology and Genetics, National Cancer Institute, Bethesda, MD 20850, USA; Charles.Matthews2@nih.gov (C.E.M.); linetm@exchange.nih.gov (M.S.L.); 7 Chinese Center for Disease Control and Prevention, Beijing 100050, China; wangyu@chinacdc.cn; 8 Office of Dietary Supplements, National Institutes of Health, Bethesda, MD 20850, USA; potischn@mail.nih.gov

**Keywords:** China, physical activity, grip strength, step count, mother–child dyads, urban–rural

## Abstract

Objectives: This paper describes the development of a physical activity questionnaire (PAQ) designed for Chinese adolescents and their mothers in urban and rural settings, and reports on results of the PAQ, pedometry, and hand grip dynamometry from the Chinese Children and Families Cohort Study pilot investigation (CFCS). Methods: As part of a pilot investigation to evaluate the feasibility to follow-up and obtain detailed nutrition, dietary, physical activity, and ultraviolet radiation (UVR) data from CFCS participants, data were collected in 2013 for 93 adolescent/mother pairs from a rural (*n* = 41) and an urban site (*n* = 52) in two provinces. Respondents were asked to wear a pedometer for seven days (Omron HJ-151), use a Takei Digital Grip Strength Dynamometer on (each hand; three trials; two separate days), and complete a 39 item, eight domain PAQ covering the past year. Self-reported physical activity (PA) was linked to metabolic equivalent of task (MET) scores in kcal/kg/hr and used to calculate METs for different domains of PA and intensity categories. Results: Compliance was high (95%) in this measurement protocol administered by health staff during a series of data collection efforts at home and local clinics or health centers. Step counts were highly variable, averaging between 5000 and 10000 per day with somewhat higher step counts in rural adolescent boys. Maximum grip strength (Kgs) was greater in children (Mean = 36.5, SE = 0.8) than mothers (Mean = 28.8, SE = 0.8) and similar in the urban (Mean = 29.6, SE = 0.6) compared to the rural (Mean = 29.6, SE = 0.5) communities overall. Grip strength, step counts, and measures of time spent in different activities or activity intensities were uncorrelated. Conclusion: Device and question-based measurement of PA and strength were readily accepted in these Chinese urban and rural populations. The PAQ on physical activity in the past year produced some plausible population averages, but individual responses suggested recall challenges. If data about specific activities are required, future studies should explore use of standardized survey questions concerning such fewer specific activities or instruments examining shorter time periods such as one, three, or seven day recalls.

## 1. Introduction

Physical activity (PA), including aerobic, muscle-, and bone-strengthening activities are linked to a wide variety of positive developmental and health outcomes in children and youth. In children under age six years, a recent review indicates strong associations of higher levels of PA with bone health and reduced risk of excessive weight gain and adiposity [1]. For youth over six years, meeting the PA guidelines of 60 min of moderate to vigorous PA (MVPA) per day contributes not only to aerobic fitness, bone health and cognitive function, but is also linked to improved biomarkers related to cardiovascular disease, diabetes, and reduced risk of such conditions in adulthood [2]. Prevalence of meeting PA guidelines in higher income countries including the US is low, with only 18% of girls and 35% of boys in high school reporting at least 60 min of PA daily [3]. Increases in obesity and reports of lower levels of PA are also emerging from low- and middle-income countries, with the most rapid increases in childhood obesity occurring in developing countries [4,5].

Valid and reliable instruments have been developed to measure diverse aspects of physical activity using several approaches including proxy and self-report, device-based measurement with pedometry and accelerometry, and measures of performance such as dynamometry, ergometry, and flexibility testing [6,7,8,9]. Current research suggests that the optimal approach to comprehensive measurement of PA involves a combination of instruments as no one approach is adequate to capture different aspects of PA relevant to health [10]. For example, device-based measures using accelerometers can capture bodily movements that are correlated with total activity levels but currently cannot measure the types of diverse activities that can be captured with self or proxy reports using standardized survey questions, 24-hour and multiday day recalls, or diaries [6,9]. Knowledge of specific behaviors can be important for intervention design and evaluation. However, relatively few studies exist that attempt to develop and evaluate a suite of PA measures using several different approaches in concert.

There is also a relative lack of validated instruments for PA measurement in many countries, including China. Self-report instruments have been assessed for children in China, Taiwan, and Hong-Kong and these have performed as well as comparable instruments in other countries. Examples in China and Hong Kong include the Physical Activity Questionnaire for Older Children (PAQ-C) for older children [11], a seven-day PA questionnaire for children [12], and the Children’s Leisure Activities Study Survey (CLASS) questionnaire [13]. A comprehensive review of PA measurement instrument validity in children in China appears to be lacking. In China, instruments validated for adults include the International Physical Activity Questionnaire (IPAQ) [14] and Shanghai PAQ [15]. China is a particularly important region for studies of physical activity and obesity because of its large population, rapid urbanization and industrialization, and the increase in obesity that has occurred in the past few decades [16]. For example, in 1985, less than 1% of Chinese youth in Shandong province had obesity but, by 2014, 17% of boys and 9% of girls had obesity based on the Working Group on Obesity in China cutpoints [17,18]. Based on self-reports, levels of physical activity are also decreasing or remaining largely unchanged in China [19,20] with substantial declines in physical fitness since 1995, especially in urban areas [21]. Growing evidence from studies set in China connects obesity and diverse health outcomes including diabetes, cardiovascular disease and cancer [22,23,24]. Addressing health outcomes related to obesity in China requires further attention to physical activity and diet, proximal determinants of energy balance, but also to weight stigma, a further cause of adverse negative psychological and physiological outcomes related to obesity [25,26,27].

In addition to understanding the prevalence and health consequences of current obesity, better understanding of patterns of obesity over the life course is an important but incompletely understood research topic. Availability of long-term longitudinal cohort studies that include details of neonatal characteristics is limited and many such cohorts are in Europe and North America. Thus, the Chinese Children and Families Cohort Study (CFCS) was launched to determine the feasibility of following-up participants of the Community Intervention Program (CIP), a study of 247,000 Chinese women who took or did not take folic acid supplementation and their offspring [28]. The CIP collected considerable data concerning maternal and offspring characteristics from periconception to birth to examine the effects of folic acid supplementation on the risk of neural tube defects (NTD), and found that supplementation during the peri-conception period significantly reduced the frequency of NTD in an area of northern China with a higher prevalence of NTD and, to a lesser, but still substantial extent, in southern China in areas with a lower baseline prevalence. About 90% of CIP subjects were re-contacted in 2000–2001 and no adverse effects of folic acid were noted among users. A new follow-up of CIP could help establish the cohort as a platform for further longitudinal study of perinatal, birth, and growth characteristics as well as long-term effects of folic acid.

In 2012–2013, the CFCS, a feasibility study assessing the possibility of locating and interviewing study participants was completed on 460 CIP mothers and 462 CIP offspring [29]. Within this feasibility study, we conducted a detailed pilot study in which we tested a newly developed physical activity questionnaire (PAQ) and protocols for collecting pedometry and hand-grip dynamometer data from 93 mothers and their adolescent children carried out in two locations in China. This study was designed to provide information concerning several aspects of PA and strength in urban and rural mother/offspring pairs and to explore the feasibility of multimodal PA assessment in the CFCS, a follow-up study of a small subset of the CIP [28,29]. In addition to establishing the feasibility of obtaining these measurements in novel settings, this study presents unusually detailed information about physical activity and strength in urban versus rural populations in China. It also provides important data to inform future larger studies on questions related to in utero exposure to folic acid and to address emerging increases in obesity and diabetes in China and elsewhere.

## 2. Materials and Methods

This paper reports on the PA portion of the Chinese CFCS which was conducted in two phases. In the first phase, methods were developed and tested to identify members of the original CIP cohort and recruit them for participation in the follow-up feasibility study [29]. In the second phase of this work, methods were developed and tested for a multiday assessment of diet, physical activity, UVR exposure, and other measures potentially related to health outcomes stemming from early life exposures to folic acid and to the development of cancer later in life. Previous papers have described the overall design of phases 1 and 2 of the Chinese CFCS [29,30] and UVR exposure methods and results [31]. In the present study we report on the feasibility of measurements and results for physical activity and strength. All phases of the CFCS protocol were approved by the Chinese provincial, city, and county health bureaus, county maternal and child health and other participating hospitals, and by the Chinese and United States (U.S.) Centers for Disease Control and Prevention and the U.S. National Cancer Institute ethics review committees (IRB #:12CN038).

In brief, a rural area in northeastern China and an urban area in south central China were selected and 93 mother–child dyads were recruited from 462 families who had participated in the CFCS feasibility study for an 8-day test of data collection methods. All data were collected from April–June 2012. Tested methods included questionnaire and device-based measurements along with collection of blood, saliva, and toenail samples. Physical activity data collection, the subject of this paper, included handgrip dynamometry, pedometry, and a past year PAQ developed for this study. Further cancer and energy balance related variables explored in this study included: (1) UVR exposure data based on a diary and dosimetry [31] and (2) dietary data from a food frequency questionnaire and 3-day food record [29,30].

Prior to data collection, a training workshop was conducted at each study center with local interviewers. The experts from China CDC led the trainings and practice sessions with the interviewers. A training manual with all procedures in detail was delivered to each interviewer.

Over the course of an eight-day study period, participants completed two handgrip dynamometry tests, wore a pedometer for seven days, and completed a past year PAQ. Interviewers interacted with participants five times, first one or two days prior to the start of the eight-day data collection window mostly at home or school and once at the township hospital. A detailed diagram of the data collection protocol is available [30]. At the first visit, after obtaining informed consent or child assent, participants provided the first of two hand grip strength measurements using a Dynamometer, and received a pedometer (OMRON HJ-151) with instructions to record step count measurements. During this visit, interviewers administered a physical activity questionnaire (PAQ) to capture self-reported PA in the past year, in all aspects of life including household activities and those for leisure, transportation, work, and school.

### 2.1. Handgrip Dynamometry

Grip strength data were collected using a Takei Digital Grip Strength Dynamometer, Model T.K.K.5401. We followed methods used in the 2011–2012 US NHANES survey [32]. Device handles were calibrated for participant hand size and participants were asked to remove their shoes and stand with feet hip width apart, holding the dynamometer in one hand down along the body so the arm was fully extended, and the dynamometer was not touching the leg. Several models of the Takei Dynamometer are known to give valid and reliable estimates of grip strength [33,34].

Participants were asked to squeeze the dynamometer three times on each hand, alternating hands and with sixty sec rest between trials. Measurements were also made on two separate days. Participants were tested on days zero and one of the protocol, with day six as a make-up day. The maximal contraction on each hand within a day was summed to yield the combined handgrip strength value used for analysis. Additionally, average values for all three trials were calculated for comparison with some past studies.

### 2.2. Pedometry

Step counts were collected with an Omron Hj-151 pedometer worn at the hip. Participants were asked to wear the pedometer all day, except while bathing, swimming, and sleeping. Participants were also asked to fill out a daily log, listing non-wear periods and their daily step counts. On study day eight, interviewers collected the pedometers and recorded step counts for each of the previous seven days. The Omron Hj-151 accurately reports step counts at prescribed and self-determined walking speeds in children and adults [35,36]. Non-wear days and days in the lowest 5% of step counts (<910 Steps/day) were excluded (~5% of the sample days) and pedometry data were missing for one urban mother and one urban female adolescent and one rural male adolescent.

### 2.3. Past Year Physical Activity Recall Instrument

We developed a past year, 39 item, eight domain physical activity recall questionnaire designed for administration to both adolescent and mother participants (Supplementary Tables S1 and S2). Domains addressed included Occupational, Caregiving, Farming, Household, Leisure/Recreational, School, Transportation, and Walking with several items per domain. Ancillary open-ended questions were asked about additional activities and stair-climbing. The instrument was developed in English and translated into Chinese by members of the study team. Back-translation and several rounds of discussion among the study team and review by team mates not involved in the translation were used to maximize comparability of the items between the English and Chinese versions. The PAQ was based on a past-year PAQ validated in a population of adults in Shanghai [15]. For the present study, Farming was added as a domain to capture activities reported in the rural as well as urban settings included in this study, and questions about activities in school were added to account for the mixed ages of participants. The PAQ involved a paper form that queried whether an activity was performed, in how many months it occurred, on average how many days per week or month it was carried out and on average how long were the activities done per day. There was no specified minimum duration for reported activities. The PAQ was administered by trained study interviewers.

Each activity from the PAQ was assigned a MET score (Supplementary Table S3) from the Adult Compendium of Physical activities [37], often the average of several similar activities from the compendium [38]. A youth compendium is also available [39], however it lacks data for many occupational activities and so we chose to use the adult compendium for both the mothers and adolescents in this study. This choice is likely to result in modest overestimates of the estimated level of energy expenditure in these adolescents because of their elevated mass-specific resting metabolic rates compared to adults. MET values for both the adolescents and adults in this study. One MET (metabolic equivalent) is defined as 1 kcal/kg/hr and is roughly equivalent to the energy cost of sitting quietly. MET scores are widely used to compare energy expenditure levels associated with activity and they adjust for average differences between body weight and resting metabolic rate. The MET scores were used to calculate time spent in sedentary, light, and moderate+ PA as well as to calculate METs per day. Note a useful discussion of MET scores is available [40]. Sedentary behavior was categorized based on reported posture (e.g., sitting), activities with MET scores > 3.0 were classifies as moderate or vigorous. We further calculated MET hours for several categories of activities including: (1) Eight domains from the PAQ, (2) sedentary, light, and moderate + vigorous intensity activities, (3) a composite of walking from multiple domains, and (4) total METs while awake with and without sedentary time included.

### 2.4. Data and Statistical Analysis

We calculated means and standard errors for measured parameters. Estimates for mothers and offspring in urban and rural settings were obtained from analysis of variance, with means comparisons using Tukey’s Honestly Significant Difference for pairwise comparisons after ANOVA or linear contrasts for comparing grouped means e.g., comparing mothers and offspring from rural areas with mothers and offspring from urban areas. We note that there are a large number of pairwise comparisons embedded in the tables. We did not adjust for multiple comparisons in this observational pilot study with a modest sample size and note that such adjustment would reduce the number of significant differences between groups but would not influence the overall patterns observed or eliminate the statistical significance of any of the ANOVAs presented. Spearman correlations were calculated for combinations of measures and between values for mothers and offspring, and between various self-reported PA measures from the PAQ with grip strength, step count, and total METS. Analyses were performed in SAS Version 9.4 or SAS JMP Version 13.0 and 14.0 (SAS Institute Inc. Cary NC, USA).

## 3. Results

Participants in this study included 41 rural/northeastern and 52 urban/southeastern mother/offspring dyads. Two rural mothers were missing either steps data or grip strength and one offspring was missing step counts resulting in a total of 38 rural dyads for analysis. (Table 1). Two urban male offspring were missing step counts. Further details concerning these dyads are available [30,31]. Briefly, over all respondents, mean age of mothers was 38.7 (Range 35–54) and offspring 15.1 (Range 14–17) years. On average, rural mothers were significantly (*p* < 0.05) older in age and had higher measured body weight and BMI compared to urban mothers (*p* < 0.05). The sample of rural mothers included three mothers >50 years old resulting in a somewhat higher mean age for the rural sample. Exclusion of these older moms reduced the mean age difference from ~3 years to ~2 years. Most of the rural mothers reported working in farming at home, while >90% of urban mothers reported employment outside the home. Mothers BMI ranged from 16.2–37.3 and offspring from 16–34.

Almost all children in the study reported attending boarding school during the week which likely results in specific patterns of physical activity such as the school-required 20-min runs each day and little or no transportation, active, or vehicular associated with attending school during the week. Additionally, some adolescent subjects reported neither going to school, nor working. Due to these responses, the percentage of those attending school and working percentages never totaled 100. Overall, we found that the design of the PAQ was not optimized for computing study subjects’ PA duration on specific days (see below), so we are cautious about interpreting details of time reported in different functional activity categories such as education or occupation.

### 3.1. Dynamometry

Maximum and average grip strength differed by urban/rural settings for mothers and by sex of offspring (Table 2). Maximum grip strength (kg) was higher (*p* < 0.05) in urban (30.1, SE = 0.7) compared to rural mothers (27.1 ± 0.85), but higher (0.05) in rural female offspring (29.0 ± 1.0) compared to urban female offspring (26.8 ± 1.2). Boys had higher grip strength than girls in both settings; average grip strength was somewhat higher among boys in rural compared to urban settings. Similar patterns were observed for average grip strength. Correlations between left and right handgrip strength and grip strength on the two measurement days ranged from 0.85 to 0.95 for mothers and offspring separately, with somewhat higher correlations for offspring than mothers. Measurements made on the second day averaged slightly higher in mothers (0.9 lbs.) and offspring of both sexes (Boys = 0.4 lbs.; Girls 0.6 lbs.).

### 3.2. Pedometry

Step counts by week and for parts of the week illustrate significantly higher greater step counts in rural male adolescents on Saturdays (Table 3) and on Saturdays and Sundays combined. Substantial variability was observed for step counts with coefficients of variation around 30–50%. Rural mothers, boys, and girls consistently recorded mores steps than their urban counterparts. In the rural area, recorded step counts were consistently higher in boys and girls compared to mothers, regardless of weekday or weekend. In the urban area, offspring recorded higher step counts than mothers on weekdays but lower counts than mothers on weekends. However, these patterns were not statistically significant except for higher step counts in rural adolescent boys on Saturdays and Saturday and Sunday combined. Maximum step counts reached 25,000+ but 90% of recorded steps per day were fewer than 13,000. Median step counts per day displayed similar patterns.

### 3.3. Physical Activity Questionnaire

Respondents reported a wide range (2–39 h per day, not including sleep) of diverse physical activities. This wide range of times reported also resulted in a large range of MET hours per day calculated based on time spent in activities and the associated Met Scores. These calculations resulted in 3-118 MET hours per day (Table 4) with a median value of 22.1 MET hrs/d. Exclusion of one subject reporting 118 MET hrs/d per day and six subjects reporting <10 MET hrs/d per day did not alter the qualitative results described below. Rural respondents had more instances of reporting >24 h of activity per day. Rural mothers reported more farming, caregiving, and household activities whereas urban mothers reported more non-farming occupational activity. Non-farming occupational activities were queried in intensity categories with examples of specific job types ranging from desk work and attending meetings to construction and mining (Supplementary Tables S1 and S2). Note we consider these results descriptive and do not report ANOVA or means comparisons.

**Figure 1:**
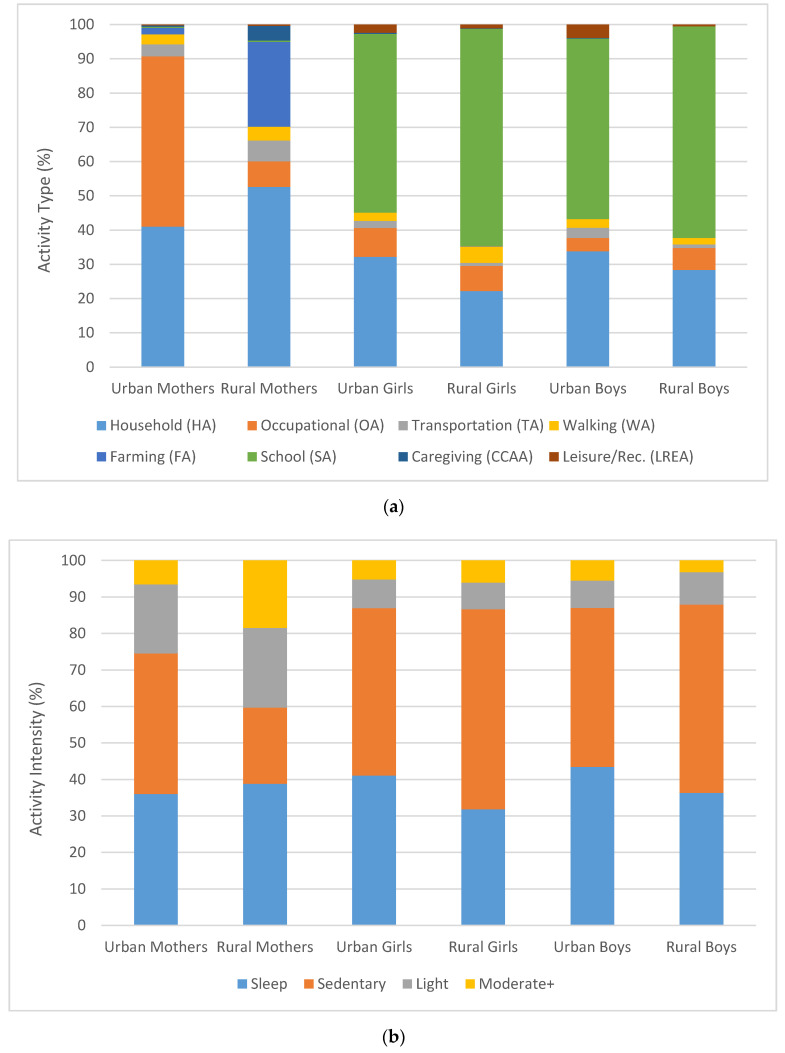
(**a**) Distribution of activities by domain for urban and rural mothers and their offspring. (**b**) Distribution of activities by intensity for urban and rural mothers and their offspring. See text for statistical details.

Among offspring, neither urban or rural youth reported participating in farming or caregiving. Rural offspring reported substantially more time in school (490–569 min) than urban offspring (312–339) minutes. Both male and female rural offspring also reported more MET hours per day than urban offspring (Table 4, although these differences were not statistically significant (*p* > 0.05)). Percent time in different activities for mothers and offspring in the urban and rural samples are illustrated in Figure 1a. Distinct activity patterns are apparent, with occupational activities dominant for urban mothers, school-based activity for children, and a mix of household and farming activities for children. Note these data are presented to illustrate the pattern of activity, we do not present statistical tests comparing these time allocations.

We also calculated reported time spent in different activity intensity categories (Figure 1b) which includes activities divided up by intensity from the household, leisure, transportation, farming, school, and occupational domains of the PAQ (Table 5). Urban mothers reported more sedentary time (8.0 Hours, SD = 1.0) than rural mothers (4.3, SD = 2.7) whereas rural offspring reported more sedentary time (Boys 10.7, SD = 5.2); Girls 12.0, SD = 6.6) than urban offspring (Boys 7.6, SD = 2.0); Girls 8.2, SD = 2.2). Note we report these estimates in hours for ease of consideration. These differences reflect the greater amount of time reported in school, particularly for rural youth. Differences between rural and urban mothers and between rural and urban offspring were statistically significant (*p* < 0.05). Estimated METs associated with sedentary, light, and moderate+ activity are shown in Table 6. These results highlight the greater amounts of sedentary time reported by rural children and the greater amount of moderate to vigorous activity reported by rural mothers (Figure 1b). Furthermore, they illustrate the large fraction of daily energy expenditure that can be accounted for by sedentary time when overall activity levels are low and sedentary time (such as sitting, watching television or video, or working on the computer) consumes a large part of the day. This can be seen by contrasting physical activity energy expenditures levels with and without sedentary activity included (Table 6).

At the time of this study, Chinees schools instituted twice a day outdoor running activity for attendees. Note that such policies do not appear to be uniform across China [41,42]. Offspring may have reported this activity under activities in the school domain or reported it under the leisure time activity domain.

### 3.4. Mother–Offspring Correlations

Correlations between mother and offspring in grip strength, step counts, and MET scores from walking-related activities, strength-related activities, light activities, and moderate to vigorous and total activities are presented in Table 7. We found significant correlations between mother and female offspring in total METs, light activity METs and walking related METS; no such correlation was observed between mother and male offspring. None of the other mother–offspring correlations were significant or near significant.

### 3.5. Correlations between Pedometry, Strength, and PAQ Measures

Device-based measures of strength and step counts and estimates of MET hours per day in different activity categories were largely uncorrelated (Table 8). MET hours per day reported in leisure and school activities were weakly associated with grip strength, consistent with the greater grip strength of offspring (who are much more likely to be in school) than mothers. Positive correlations between MET hours associated with light activity, moderate+ activity as well as occupational, farming, household, transportation and walking MET hrs/d and total MET hrs/d reflect the fact that these activities comprise total MET hrs/d. Modest sample sizes preclude a more detailed analysis by region and in mothers and offspring although there was some evidence that correlations between strength, step counts and MET hours were stronger in boys (not shown).

We also summed time spent in walking or strength related activities in specific elements of the main activity domains and calculated MET hrs/d for these summed times. Walking activities occurred in the Walking domain, but were also queried as part of farming, school, and occupation activities (Supplementary Tables S1 and S2). MET hrs/d based on this measure of self-reported walking were uncorrelated with step counts (*p* = 0.08) and MET hrs/d based on strength related activities were uncorrelated with grip strength (*p* = −0.02). Separate analyses by generation and sex resulted in qualitatively similar results although sample sizes were small (not shown).

## 4. Discussion

This study is one of a modest number of efforts to explore multiple measures of physical activity and performance in Chinese children and families [12,18,21,43,44]. As far as we can tell, it is the first such study to describe rural and urban differences in mother–child dyads in China with respect to multiple PA measures. This is a particularly timely and important study because chronic diseases are increasing globally, and the World Health Organization has recently released a global action plan for physical activity [45]. The study demonstrates good performance and acceptability of device-based measurement via pedometry and grip strength and highlights some challenges in the use of interviewer-administered questionnaire to capture self-reported past year physical activity in multiple domains. Notably, average daily hours of activity reported in the PAQ were highly variable, ranging from 2 to 39 h, not including sleep, and correlations with measured step counts and strength were weak or absent. Together these results support the use of pedometry and dynamometry measurements and suggest that further work is needed to select appropriate instruments to measure diverse PA behaviors in urban and rural settings for both adults and adolescents. Simpler questionnaires focusing on specific activities or use of multiple previous day recalls are potential solutions to the cognitive challenges of self-reporting [6,9].

As in past studies, grip strength in both mothers and offspring was highly repeatable and showed differences by age and gender [32]. In this sample of Chinese mothers aged ~40 and offspring aged ~15 years, boys had grip strengths 36–39% higher than girls and mothers’ grip strength was similar to that in adolescent daughters. In a large sample of children from the United States, grip strength was 29% higher in 15 year-old boys compared to girls [32]. Somewhat lower grip strength then found here has been reported for Chinese adolescents using measurements obtained using the Jamar Hydraulic Hand Evaluation Kit [44]. Cross-instrument validation studies in China could be useful to allow data pooling.

We report similar grip strength in urban and rural samples, as did one prior study of urban and rural 10–12 year old Chinese children [46]. One puzzling observation is that grip strength was somewhat higher in urban compared to rural mothers despite more time spent farming and in household activities. This may reflect differences in age, type of occupation or farming activities performed, or nutritional status in our samples. Muscle strength is emerging as an independent (from Aerobic Fitness) correlate of health and health outcomes across the life course [47]. Our results further support the feasibility of measuring grip strength in both urban and rural populations in China and provide data on adolescent and maternal grip strength in two Chinese contexts.

Step counts have been the subject of considerable research as correlates of total physical activity and as a relatively easy way to measure aspect of the most common form of physical activity (walking) in many countries. Much attention has been paid to a target count of 10,000 steps per day and to the benefits of moderate and vigorous activity [2]. However, recent epidemiological analyses of associations between activity and mortality suggest that fewer steps and light activity are associated with reduced mortality rates in older adults and may also have benefits in younger populations [2,48,49,50]. In this study, pedometry compliance was high and wear times largely appeared to encompass the entire day. Rural male adolescents had higher step counts on Saturdays than urban adolescents. We lack data to explain this difference—future studies could consider use of 24-hour recalls to better describe behaviors accounting for this difference in step counts. Rural offspring had higher step counts than urban offspring and average step counts per day were close to 10,000 for rural boys but these differences were not significant. This could reflect mandatory PA (running) at boarding schools or relatively larger distances and lower building density in rural areas. However, there was substantial unexplained variation between individuals and more contextual data may be needed to account for differences in step counts. Note we cannot meaningfully assess repeatability of the pedometry sample because day to day variability in steps likely represents differences in behavior rather than measurement characteristics of the devices.

The step counts obtained in our study in urban areas were somewhat lower than other studies in China. One study of older adults (~65 years old) in Guangzhou, using the Yamax SW-200 pedometer, reports ~10,000 steps per day in both men and women [43] and a study of Chinese children aged 9–12 in Hong Kong using the New Lifestyles NL-800 piezoelectric pedometer reported daily step counts of about 9000 for boys and 8000 for girls during week three of a three week study [51]. Ling noted a significant decline in step counts from week one to week three, suggesting the estimated step counts in our study could also be overestimates of normal step counts due to reactivity. In our study, urban mothers were more likely to report factory work which may account for the generally lower step count. Nevertheless, as with dynamometry, pedometry appears to be a viable and reasonable data collection method for an important element of physical activity.

Our results indicated good performance by the Omron HJ-151 pedometer. However, this device is no longer manufactured. Similar devices are available for under $20 in US currency. Newer models have not always been validated and this remains a problem when using consumer-oriented products for research purposes. On the other hand, most consumer pedometers are based on the similar underlying technology and performance is often comparable between different devices [52,53]. Nevertheless, caution should be exercised in comparing results from devices made by different manufacturers, from unvalidated new models, and from devices that have never been validated. It may be useful to conduct a validation study comparing step counts obtained from a new device or in a population that might display different gait characteristics to manually counted steps in self-selected or treadmill-controlled speeds to ensure that valid estimates of step counts are obtained. Such studies are not resource intensive [52].

Results from the PAQ questionnaire showed some evidence of cognitive challenges related to past year recalls [6,9] and some evidence that the average reported times in different activity categories and different intensities reflect likely differences between parents and offspring and between urban and rural parents. The wide range of average times per day reported suggests difficulty in recalling absolute amounts of activity in different categories whereas some of the reported patterns of activity in different domains are consistent with differences in context and between generations. For example, urban mothers reported more time working and rural mothers more time farming. The greater reported amount of farming resulted in higher estimates of MET hrs/d for rural mothers. Interestingly, rural mothers also had higher BMIs than urban mothers. Further data on diet and body composition could help explain this pattern.

Urban children spent less time in school and less time in sedentary activities than rural children, consistent with the observation that almost all the rural children boarded at school during the week whereas fewer urban children did so. Lastly, while the estimated MET hours per day (27–40) are reasonable, the weak correlations between grip strength and step counts versus Met scores associated with different types of activities provide further evidence of potential challenges in accurate responses to the PAQ. Weak correlations between time spent walking estimated from the PAQ and step counts could also reflect the temporal mismatch between the data collected with these instruments. However, device-based measurements do show relatively high intraclass correlations over 6–12 month periods in some studies [54]. Steps were collected for one week whereas the PAQ reflects an average over the past year. Only leisure and school related MET scores were associated with grip strength, consistent with the idea that more time in sports and in school related sports could be associated with strength, but these associations were still weak.

Key strengths of the study include the extensive effort to test the performance and feasibility of instrument and self-report measurement of PA in mothers and offspring from two distinct settings in China. Such detailed analyses of urban and rural differences and inclusion of cross-generational comparisons are important because of rapid increases in sedentary behavior, obesity, diabetes, and urban–rural disparities in China [41]. The results reported here and in related papers concerning UVR exposure, diet and other variables [29,30,31] also provide valuable guidance both for a potential follow up to the 1993–1995 Community Intervention Program (CIP) and for PA measurement in other cohort studies in China. Additionally, high response rates and largely complete data reflect the investment on standardized training of interviewers and ongoing data quality monitoring during data collection.

Limitations of this study include the modest sample size and lack of a direct validation component to this study of different measures of physical activity and strength. The devices used here captured step counts and grip strength, aspects of performance that were not directly captured in the PAQ. Nevertheless, weak correlations between these instrument-based measurement and aspects of self-reported physical activity suggest considerable measurement error in our PAQ instrument. Past year physical activity recalls involve significant cognitive challenges related to recall of diverse and sometimes intermittent or seasonal activities [6] and these challenges can be even more salient in youth [9]. Seasonal aspects of farming and school attendance may have made the past year approach particularly challenging in this population. Future studies should consider use of a simpler instrument for measuring PA such as IPAQ [43], the Chinese Health and Nutrition Survey [55] or to explore the use of multiple one, three, or seven day physical activity recalls. Like the PAQ described here, shorter duration recalls can capture rich information concerning type and context of activities, but they have better measurement properties and reduced error because it is far easier for people to recall what they did yesterday or over the past three or seven days than over the past year [56,57]. A limitation of this approach is the greater respondent burden associated with completing the two or more 24-hour recalls required to estimate within and between subject variability in PA. Each recall takes 15 or more minutes to complete. We are not aware of a device-based Chinese language 24-hour PA recall developed although time use surveys have been carried out in China and many other countries [58].

A further weakness of this study is the use of MET scores from the Adult Compendium to calculate MET values for the adolescent participants. A youth compendium is available [39], however, many activities common in this Chinese population are missing from the Youth Compendium. Efforts to obtain more precise estimates of MET scores for typical activities in Chinese youth in urban and rural areas would be useful. Nevertheless, errors associated with the use of the adult MET score are likely to be modest because the adolescents in this study were old enough that their MET values for different activities are similar to those in adults [59].

More broadly, these results may contribute to understanding trends in obesity and metabolic health in China and inform the design of more effective individual and policy level interventions. As mentioned above, self-reported levels of physical activity are decreasing or unchanged in recent years and prevalence of obesity is increasing [20,60]. Knowledge of specific factors that are driving the decline in PA in youth is incomplete. It is likely that there are multiple factors, including maternal characteristics, perinatal, and neonatal characteristics, and changing aspects of the built, natural, social and digital environments influencing trends in physical activity and obesity in China. Better understanding of the influence of these factors over the whole developmental trajectory, as well as during critical windows of susceptibility [61,62], could contribute to the design of more precise measurement and effective intervention strategies tailored to the environment in different settings, including urban and rural locations in China and other countries.

## 5. Conclusions

Results of this study support the utility and feasibility of using pedometry and grip strength dynamometry in urban and rural Chinese mothers and adolescent offspring. These results require confirmation in larger studies, with an emphasis on additional investigations of contextual factors, including, urban vs. rural differences and their association with physical activity, BMI, and attitudes towards obesity, and development of improved approaches to capturing specific activities.

## Figures and Tables

**Table 1:** Participant characteristics. Further details are available [30,31].

	Urban			Rural			*p*-Values ^5^
	Mother	Offspring		Mother	Offspring		
		Male	Female		Male ^2^	Female	
N	52	33	18	39	13	27	
Age (years) ^1^	38.7 ^a^ (2.2)	15.2 ^b^ (0.7)	15.1 ^b^ (0.7)	41.6 ^c^ (4.8)	15.0 ^b^ (0.4)	15.4 ^b^ (0.7)	<0.0001
Height (m) ^1^	1.57 ^a^ (0.05)	1.71 ^b^ (0.05)	1.62 ^c^ (0.04)	1.57 ^a^ (0.05)	1.70 ^b^ (0.03)	1.6 ^c^ (0.06)	<0.0001
Weight (kg) ^1^	55.4 (8) ^b^	63.7 (11) ^a^	54.9 (10) ^b^	63.1 (9) ^a^	60.4 (10) ^a,b^	58.0 (9) ^a,b^	<0.0001
BMI (kg/m^2^) ^1^	22.4 (2.9) ^b^	21.9 (3.8) ^b^	21.0 (3.8) ^b^	25.6 (3.6) ^a^	21.0 (3.5) ^b^	22.3 (3.2) ^b^	<0.0001
	M + F Combined			M + F Combined			
Attending School (%)	5.8%	88.5%	2.6%	97.5%			
Working (%) ^3^	92.3%	13.5%	23.1% ^4^	22.5%			

^1^ Mean, Standard Deviation; m = meters, kg = kilograms; Means with different superscripts are significantly different (*p* <0.05); ^2^ There was one child here with missing covariate data, but non-missing gender; ^3^ Some subjects who report both going to school and working and some subjects who report neither going to school, nor working. Due to these responses, the sum of the attending school and working percentages is never 100; ^4^ Non-working rural mothers largely did farm work at home and/or raised animals for income; ^5^ *p*-value for full ANOVA model including region, demographic group, and interaction terms. Means labeled with different superscripts (^a,b,c^) are significantly different with *p* < 0.05.

**Table 2:** Grip strength of Chinese mothers and offspring measured with the Takei Digital Grip Strength Dynamometer, Model T.K.K.5401.

	Urban			Rural			*p*-Values ^3^
	Mother	Offspring		Mother	Offspring		
		Male	Female		Male	Female	
N	52	33	19	39	13	27	
Maximum Grip Strength (Kg) ^1^							
Left Hand ^2^	28.3 (4.8) ^b^	41.4 (6.9) ^a^	24.9 (3.4) ^b^	25.5 (4.9) ^b^	41.7 (6.3) ^a^	27.5 (5.5) ^b^	<0.0001
Right Hand	29.8 (4.9) ^b^	43.8 (6.0) ^a^	26.8 (3.9) ^b^	26.7 (4.9) ^b^	45.5 (7.2) ^a^	28.7 (5.9) ^b^	<0.0001
Combined	58.1 (9.4) ^b^	85.2 (12.4) ^a^	51.7 (7.2) ^b^	52.2 (9.4) ^b^	87.2 (13.1) ^a^	56.1 (11.2) ^b^	<0.0001
Average Grip Strength (Kg) ^1^							
Left Hand ^2^	26.1 (4.5) ^b^	37.8 (6.3) ^a^	22.7 (3.5) ^b^	23.3 (4.8) ^b^	39.2 (6.5) ^a^	25.0 (4.9) ^b^	<0.0001
Right Hand	27.6 (4.7) ^b^	40.2 (5.7) ^a^	24.1 (3.9) ^b^	24.5 (5.3) ^b^	42.9 (7.2) ^a^	26.5 (5.8) ^b^	<0.0001
Combined	53.7 (9.0) ^b^	78.0 (11.6) ^a^	46.8 (7.3)^b^	47.83 (9.8) ^b^	82.1 (13.3) ^a^	51.5 (10.6) ^b^	<0.0001

^1^ Kg = kilogram; ^2^ Mean, Standard Deviation; ^3^ *p*-value for full ANOVA model including region, demographic group, and interaction terms. Means labeled with different superscripts (^a,b^) are significantly different with *p* < 0.05.

**Table 3:** Daily step counts of Chinese parents and offspring measured with the Omron HJ-151 Pedometer.

	Urban			Rural			*p*-Values ^2^
	Mother	Offspring		Mother	Offspring		
		Male	Female		Male	Female	
N	51	33	18	39	12	27	
Average Step Count per day ^1^							
Total Week	7327 (3264)	7275 (3160)	7860 (2012)	7506 (3717)	9507 (3070)	8040 (2434)	0.301
Weekday	7419 (3457)	7813 (3639)	8065 (2970)	7584 (3722)	9423 (3226)	8131 (2440)	0.557
Saturday	7375 (4295) ^a,b^	4935 (4162) ^b^	6299 (3358) ^a,b^	7605 (5676) ^a,b^	10,322 (4311) ^a^	7980 (3469) ^a,b^	0.007
Sunday	6816 (3460)	5493 (5189)	5595 (3743)	7021 (4586)	9118 (2526)	7640 (5748)	0.136
Weekend	7096 (3340) ^a,b^	5214 (4141) ^b^	5947 (2390) ^a,b^	7312 (4372) ^a,b^	9720 (3063) ^a^	7810 (3989) ^a,b^	0.006

^1^ Mean, Standard Deviation; ^2^ *p*-value for full ANOVA model including region, demographic group, and interaction terms. Means labeled with different superscripts (^a,b^) are significantly different with *p* < 0.05. If the full model was not significant, means comparisons were not performed.

**Table 4:** Reported time spent in different activity domains by Chinese parents and offspring based on the Chinese Children and Families Cohort Study Physical Activity Questionnaire.

	Urban			Rural		
Activity Domain	Mother	Offspring		Mother	Offspring	
Mean Minutes (SD) ^1^		M	F		M	F
N	52	33	19	39	13	27
Occupational (OA)	397 (176)	23 (105)	53 (160)	56 (174)	51 (129)	66 (192)
Caregiving (CCAA)	3 (11)	1 (7)	2 (8)	32 (126)	0 (0)	1 (4)
Farming (FA)	15 (41)	0 (0)	0 (0)	186 (188)	0 (1)	1 (3)
Household (HA)	327 (133)	200 (146)	203 (129)	395 (213)	225 (243)	199 (241)
Leisure/Rec. (LREA)	2 (12)	23 (35)	15 (24)	3 (19)	4 (7)	10 (20)
School (SA)	3 (15)	312 (164)	329 (170)	3 (19)	490 (297)	569 (216)
Transportation (TA)	28 (22)	18 (21)	13 (12)	46 (44)	8 (13)	8 (10)
Walking (WA)	23 (30)	15 (14)	15 (16)	30 (43)	15 (18)	43 (64)
Mean hours Reported	800 (150)	593 (133)	631 (116)	750 (391)	792 (374)	897 (499)
Hours/day (Range) ^2^	5–17	6–15	8–14	3–39	2–24	4–39
MET hrs/d per day	25.5 (7.99)	20.4 (5.87)	20.5 (5.82)	33.0 (21.90)	25.0 (11.82)	30.1 (16.14)
MET hrs/d (Range)	9–51	10–36	11–41	5–118	3–44	9–66

^1^ SD = Standard Deviation. ^2^ Not including sleep time.

**Table 5:** Reported hours per day spent in different activity intensities by Chinese parents and offspring based on the CCFC Physical Activity Questionnaire.

Hours Per Day ^1^	Urban			Rural			*p*-Value ^2^
	Mother	Offspring		Mother	Offspring		
		M	F		M	F	
N	52	33	19	39	13	27	
Sleep	7.5 (1.0)	7.6 (1.0)	7.3 (0.9)	8.0 (1.9)	7.5 (1.0)	7.0 (2.4)	0.2246
Sedentary	8.0 (3.7) ^a^	7.6 (2.0) ^a^	8.2 (2.2) ^a^	4.3 (2.7) ^c^	10.7 (5.2) ^a,b^	12.0 (6.6) ^b^	0.0001
Light	3.9 (3.0) ^a,b^	1.3 (1.8) ^c^	1.4 (0.9) ^c^	4.5 (3.1) ^a^	1.8 (2.3) ^b,c^	1.6 (2.2) ^c^	0.0001
Moderate plus	1.4 (2.2) ^a^	1.0 (0.8) ^a^	0.9 (2.1) ^a^	3.8 (3.9) ^b^	0.7 (0.5) ^a^	1.3 (1.4) ^a^	0.0001
Vigorous							
Total Hours	20.8 (2.7) ^a,b^	17.5 (2.2) ^b^	17.9 (2.1) ^a,b^	20.5 (6.4) ^a,b^	20.7 (6.6) ^a,b^	20.9 (9.2) ^b^	0.0076

^1^ Mean, Standard Deviation; ^2^ *p*-value for full ANOVA model including region, demographic group, and interaction terms. Means labeled with different superscripts (^a,b,c^) are significantly different with *p* < 0.05. If the full model was not significant, means comparisons were not performed.

**Table 6:** Metabolic equivalent of task (MET) hrs/d from different activity intensities by Chinese mothers and offspring based on the CCFC Physical Activity Questionnaire.

Activity Intensity ^1^	Urban			Rural			*p*-Value ^2^
	Mothers	Offspring		Mothers	Offspring		
		M	F		M	F	
N	52	33	19	39	13	27	
Sleep	7.1 (1.0)	7.2 (0.9)	7.0 (0.8)	7.6 (1.8)	7.2 (0.9)	6.6 (2.3)	0.2246
Sedentary	11.0 (5.2) ^a^	12.6 (3.6) ^a^	13.7 (4.0) ^a,b^	5.8 (3.9) ^d^	18.5 (8.8) ^b,c^	21.3 (10.0) ^c^	0.0001
Light	9.0 (7.3) ^a^	2.6 (4.4) ^b^	2.7 (2.0) ^b^	9.7 (7.0) ^a^	3.2 (3.5) ^b^	3.0 (3.9) ^b^	0.0001
Moderate plus	5.5 (9.2) ^a^	5.2 (276) ^a^	4.0 (7.8)^a^	17.5 (18.3) ^b^	3.2 (2.8) ^a^	5.7 (6.1)^a^	0.0001
Vigorous							
MET hrs/d (Awake)	25.5 (8.0)^a,b^	20.4 (5.9) ^b^	20.5 (5.8) ^b^	33.0 (22.0) ^a^	25.0 (11.8) ^a,b^	30.1 (16.1) ^a,b^	0.0008
MET hrs/day	14.5 (10.7) ^a^	7.8 (5.8) ^a^	6.7 (7.7)^a^	27.2 (20.8) ^b^	6.4 (4.4) ^a^	8.7 (9.6) ^a^	0.0001
(Non-sedentary)							

^1^ Mean, Standard Deviation; ^2^ *p*-value for full ANOVA model including region, demographic group, and interaction terms. Means labeled with different superscripts (^a,b,c^) are significantly different with *p* < 0.05. If the full model was not significant, means comparisons were not performed.

**Table 7:** Mother–offspring correlations for grip strength (combined maximum), steps per day, MET hrs/d per day based on strength related, walking, light and moderate activity categories.

Variable ^1^	Mother vs.		
	Male Offspring	Female Offspring	Combined
Grip Strength	0.27	−0.06	0.14
Step Count	0.28	0.09	0.18
Strength (METS)	0	0.30	0.04
Walking (METS)	0.01	**0.46**	0.16
Light METS	0.03	**0.44**	0.16
MVPA METS	−0.12	0.24	0.12
Total METS	−0.06	**0.54**	**0.40**
Non-Sedentary METS	−0.14	0.43	0.24

^1^ Correlations include all participants (*n* = 91); Bold correlations are statistically significant with *p* < 0.05.

**Table 8:** Correlations between device-based measures (maximum grip strength in kilograms and average daily step count) and MET hours (per day, in different activity categories and from light or moderate+ activity) based on self-reported physical activity over the past year from PAQ for all participants combined.

Measures	Grip Strength ^1^	Step Count	Total METS	Non-Sedentary
				METS
Grip Strength (Kgs)	1	0.13	−0.1	−0.18
Step Count (Daily)	0.12	1	0.12	0.11
Physical Activity Questionnaire				
MET hrs/Day (METS)	−0.1	0.12	1	**0.85**
Non-Sedentary Met hrs/Day	−0.18	0.11	**0.85**	1
Light Activity (METS)	−0.24	0.12	**0.46**	**0.6**
Moderate/Vigorous (METS)	−0.09	0.13	**0.8**	**0.9**
Occupational (OA)	−0.12	0.03	**0.42**	**0.45**
Caregiving (CCAA)	−0.1	−0.09	0.09	0.14
Farming (FA)	−0.2	0.01	**0.5**	**0.65**
Household (HA)	−0.2	−0.03	**0.6**	**0.56**
Leisure/Rec. (LREA)	**0.32**	0.09	0.12	0.01
School (SA)	**0.29**	0.14	0.08	−0.27
Transportation (TA)	−0.22	0.05	**0.27**	**0.44**
Walking (WA)	−0.09	0.08	**0.32**	0.23

^1^ Correlations include all participants (*n* = 91); Bold correlations are statistically significant with *p* < 0.05.

## Supplementary Materials

The following are available online at https://www.mdpi.com/1660-4601/17/17/6202/s1, Table S1 China-US Collaboration Project on Chinese Children and Families Cohort Study Physical Activity Questionnaire (English Version); Table S2 China-US Collaboration Project on Chinese Children and Families Cohort Study Physical Activity Questionnaire (Chinese Version), and Table S3 MET Scores for the Activities in the PAQ.
