# Supplementary material for: Physical Activity, Step Counts, and Grip Strength in the Chinese Children and Families Cohort Study
Source: Int J Environ Res Public Health. 2020 Aug 26;17(17):6202. doi: 10.3390/ijerph17176202 (PMC7504127; doi:10.3390/ijerph17176202)
Supplement: Supplementary file 1 [file ijerph-17-06202-s001.pdf]

Supplementary Table S1. China-US Collaboration Project on Chinese Children and Families Cohort Study Physical Activity Questionnaire (English Version)

**Participant**	Name：________ Phone number：________ Address:________________ **ID:**□□□□□□□□□□□□ (Label)
**Interviewer**	Name:
**Date**	Date：_______ (yyyymmdd) Day of week__ |_|_|_|_|_|_|_|_|_| Starting time：___ (H):___ (M) |__|__

In the next several pages, we will be asking you about your physical activity patterns over the past year (last 12 months) including household, walking, transportation, farming, occupational/school, recreational, and volunteer activities. For each activity (listed below), please tell me if you did it in the past year. If you did, please tell me 1) how many months per year, 2) how many days per month or per week, and 3) how many hours and minutes per day you did each activity.

Although some examples for each type of activities are given in the questionnaire, all activities that you do often may be NOT included. Therefore, the definitions of different intensity of activity are given as the follows. Please include all the activities with the same intensity into each type of your activities when you recall.

Light activities refer to activities that take light physical effort with breath as normal.

Moderate activities refer to activities that take moderate physical effort and make you breathe somewhat harder than normal.

Vigorous or hard activities refer to activities that take hard physical effort and make you breathe much harder than normal.

1. In the past year, have you

[1] not been in school and not had a job (Answer Part A and D）

[2] been in school and not had a job (Answer Part A, B and D)

[3] been working and not been in school (Answer Part A, C and Part D)

[4] studied for a while, and then is working or farming after quitting school /suspending school/graduation (Answer Part A, B, C and D).

Part A: Activities including: Ⅰ- Household chores; Ⅱ- Walking; Ⅲ- Transportation; Ⅳ- Caring for Children, Adults, or Pets; Ⅴ- Leisure, Recreational, and Exercise Activities, and Ⅵ- Farming activities

Ⅰ. Household Activity

	Did you participate in this activity? 1 Yes; 2 No	How many months did you do this activity per year?	How many days on average did you do this activity per month or per week? |__|__|Day/Mon / |__|day/Week	How long (H:M) on av[illegible] did you do this activi[illegible] day? |_||_|:|_|_|
1. **Sitting**: Watching TV or VCR/ VCD/ DVD	|__|	|__|__|	|__|__|/|__|	|__|__|:|__|__|
2. **Sitting**: Using computer (not at work)/school, playing video games	|__|	|__|__|	|__|__|/|__|	|__|__|:|__|__|
3. **Sitting**: Eating, reading. knitting, sewing, chatting with family or friends, playing cards, chess or Majiang.	|__|	|__|__|	|__|__|/|__|	|__|__|:|__|__|
4. **Light chores:** Cooking, washing dishes, cleaning up, laundry, dusting, shopping (time in store, not including time spent on the way), light gardening.	|__|	|__|__|	|__|__|/|__|	|__|__|:|__|__|
5. **Moderate to vigorous effort**: Heavily cleaning up rooms (brushing floor, cleaning windows, vacuuming, and washing floor, cleaning yard), washing a car.	|__|	|__|__|	|__|__|/|__|	|__|__|:|__|__|
6. **Sleeping**	|__|	|__|__|	|__|__|/|__|	|__|__|:|__|__|

Ⅱ. Walking

	Did you participate in this activity? 1 Yes; 2 No	How many months did you do this activity per year?	How many days on average did you do this activity per month or per week? |__|__|Day/Mon / | |day/Week	How long (H:M) on average did you do this activity each day? |_||_|:|_|_|
1. Walking to and from work/school, (including walking to and from bus station)	|__|	|__|__|	|__|__|/|__|	|__|__|:|__|__|
2. Out (Shopping/Chores): walking to and from household chores,	|__|	|__|__|	|__|__|/|__|	|__|__|:|__|__|

including shopping (not including time in the store), visiting friends.				
3. Walking during leisure time (not at work/school, not walking dog or birds)	|__|	|__|__|	|__|__|/|__|	|__|__|:|__|__|

Ⅲ. Transportation Activities (related to work or school, household chores and travel)

	Did you participate in this activity? 1 Yes; 2 No	How many months did you do this activity per year?	How many days on average did you do this activity per month or per week? |__|__|Day/Mon / | |day/Week	How long (H:M) on average did you do this activity each day? |_||_|:|_|_|
1. Riding bike	|__|	|__|__|	|__|__|/|__|	|__|__|:|__|__|
2. Driving motorcycle/scooter, car	|__|	|__|__|	|__|__|/|__|	|__|__|:|__|__|
3. Riding in a motorcycle/scooter, car, taxi, bus, subway, School bus.	|__|	|__|__|	|__|__|/|__|	|__|__|:|__|__|

Ⅳ. Caring for Children, Adults, or Pets

	Did you participate in this activity? 1 Yes; 2 No	How many months did you do this activity per year?	How many days on average did you do this activity per month or per week? |__|__|Day/Mon / | |day/Week	How long (H:M) on average did you do this activity each day? |_||_|:|_|_|
1. **Light effort:** bathing, feeding, playing with children (reading, drawing, toys)	|__|	|__|__|	|__|__|/|__|	|__|__|:|__|__|
2. **Moderate effort:** Lifting and carrying, pushing wheelchair or stroller, playing active game with children	|__|	|__|__|	|__|__|/|__|	|__|__|:|__|__|
3. **Caring for pets**: walking dogs, bird and cleaning pets:	|__|	|__|__|	|__|__|/|__|	|__|__|:|__|__|

Ⅴ. Leisure, Recreational, and Exercise Activities (not at work or at school)

<table>
<tr><th></th><th>Did you participate in this activity?
1 Yes; 2 No</th><th>How many months did you do this activity per year?</th><th>How many days on average did you do this activity per month or per week?
|__|__|Day/Mon / |
|day/Week</th><th>How long (H:M) on average did you do this activity each day?
|_||_|:|_|_|</th></tr>
<tr><td>1. Tutorial class out school on language, math, English, drawing, and chess (Sitting - reading, writing, speaking)</td><td>|__|</td><td>|__|__|</td><td>|__|__|/|__|</td><td>|__|__|:|__|__|</td></tr>
<tr><td>2. Tutorial class out school on music playing , (Sitting or standing)</td><td>|__|</td><td>|__|__|</td><td>|__|__|/|__|</td><td>|__|__|:|__|__|</td></tr>
<tr><td>3. Mind/Body exercises with light effort: Tai Chi, Mulan, sword dancing, fan dancing</td><td>|__|</td><td>|__|__|</td><td>|__|__|/|__|</td><td>|__|__|:|__|__|</td></tr>
<tr><td>4. Dancing (including Tutorial class out school) social or folk dancing</td><td>|__|</td><td>|__|__|</td><td>|__|__|/|__|</td><td>|__|__|:|__|__|</td></tr>
<tr><td>5. Sports- moderate effort: bowling, billiards, table tennis</td><td>|__|</td><td>|__|__|</td><td>|__|__|/|__|</td><td>|__|__|:|__|__|</td></tr>
<tr><td>6. Sports- vigorous effort: basketball, soccer, badminton, tennis</td><td>|__|</td><td>|__|__|</td><td>|__|__|/|__|</td><td>|__|__|:|__|__|</td></tr>
<tr><td>7. Conditioning exercises- moderate effort: low impact aerobics, slow swimming, roller skating, yoga.</td><td>|__|</td><td>|__|__|</td><td>|__|__|/|__|</td><td>|__|__|:|__|__|</td></tr>
<tr><td>8. Conditioning exercises- vigorous effort: step aerobics, high intensity and long time lasting swimming, jogging, climbing hills, and rope jumping</td><td>|__|</td><td>|__|__|</td><td>|__|__|/|__|</td><td>|__|__|:|__|__|</td></tr>
<tr><td>9. Strengthening exercises: lifting body, lifting weights, strength training</td><td>|__|</td><td>|__|__|</td><td>|__|__|/|__|</td><td>|__|__|:|__|__|</td></tr>
</table>

Ⅵ. Farming activities

<table>
<tr><th></th><th>Did you participate in this activity?
1 Yes; 2 No</th><th>How many months did you do this activity per year?</th><th>How many days on average did you do this activity per month or per week?</th><th>How long (H:M) on average did you do this activity each day?
|_||_|:|_|_|</th></tr>
</table>

<table>
<tr><td></td><td></td><td></td><td>|__|__|Day/Mon / |
|day/Week</td><td></td></tr>
<tr><td>1. Sitting: knitting, Shelling peas, shucking corn, embroidery,</td><td>|___|</td><td>|___|___|</td><td>|___|___|/|___|</td><td>|___|___|:|___|___|</td></tr>
<tr><td>2. Slow / intermittent walking (lasting less than 5 minutes at a time, or standing very light farming: watering crops, feeding animals</td><td>|___|</td><td>|___|___|</td><td>|___|___|/|___|</td><td>|___|___|:|___|___|</td></tr>
<tr><td>3. Standing – Moderate farming: weeding, spraying insecticide, trimming, sowing, fertilizing, spading, digging, feeding animals, fishing.</td><td>|___|</td><td>|___|___|</td><td>|___|___|/|___|</td><td>|___|___|:|___|___|</td></tr>
<tr><td>4. Standing – hard farming : manual harvesting, chopping , cleaning poultry house and barn</td><td>|___|</td><td>|___|___|</td><td>|___|___|/|___|</td><td>|___|___|:|___|___|</td></tr>
</table>

B. Activities at school

In the past year, have you studied at school? [1] Yes (answer question 1-7 in the following table) [2] no (skip to part EIGHT)

	**Did you participate in this activity? 1 Yes; 2 No**	**How many months did you do this activity per year?**	**How many days on average did you do this activity per month or per week? |_|_|Day/Mon / | |day/Week**	**How long (H:M) on average did you do this activity each day? |_||_|:|_|_|**
1. **Sitting:** listening, reading, writing, drawing, using computer, hand work, sitting in the lab	|_|	|_|_|	|_|_|/|_|	|_|_|:|_|_|
2. **Slow / intermittent walking** (lasting less than 5 minutes at a time) in the classroom or standing with light activity, like chatting with students, lab	|_|	|_|_|	|_|_|/|_|	|_|_|:|_|_|
3. **Standing**: walking from classroom to classroom, cleaning classroom or schoolyard	|_|	|_|_|	|_|_|/|_|	|_|_|:|_|_|
4. **Sports - Light effort**: radio calisthenics, warming up and cooling down activities in the physical education classes	|_|	|_|_|	|_|_|/|_|	|_|_|:|_|_|
5. **Sports - moderate effort**: Wushu, ping-pong, kicking shuttle cock, dancing, volleyball	|_|	|_|_|	|_|_|/|_|	|_|_|:|_|_|
6. **Sport - hard effort**: long-distance run, shuttle run, rope jumping, basketball, football, badminton, high intensity aerobics.	|_|	|_|_|	|_|_|/|_|	|_|_|:|_|_|
7. **Strengthening exercises:** Push-up, sit-up, pull-up, throwing	|_|	|_|_|	|_|_|/|_|	|_|_|:|_|_|

C. **Occupational Activities (including volunteer activities)**

In the past year, have you had a job? [1] Yes (answer question 1-4 in the following table) [2] no (skip to part NINE)

	**Did you participate in this activity? 1 Yes; 2 No**	**How many months did you do this activity per year?**	**How many days on average did you do this activity per month or per week? |__|__|Day/Mon / | |day/Week**	**How long (H:M) on average did you do this activity each day? |_||_|:|_|_|**
1. **Sitting work** : reading, writing, desk/computer work, sitting in meetings and labs.	|__|	|__|__|	|__|__|/|__|	|__|__|:|__|__|
2. **Slow / intermittent walking** (lasting less than 5 minutes at a time, or standing very light work, like a store clerk, Xeroxing, filing,	|__|	|__|__|	|__|__|/|__|	|__|__|:|__|__|
3. **Standing – Moderate wor**k: waitress, healthcare, cleaning, massaging, dancing teacher and others carrying items weighing less than 25 kg.	|__|	|__|__|	|__|__|/|__|	|__|__|:|__|__|
4. **Standing – hard work:** like lifting, carrying, or unloading items weighting greater than 25 kg, often done by construction workers, truck drivers, movers, delivery persons, mining.	|__|	|__|__|	|__|__|/|__|	|__|__|:|__|__|

D. Other questions

1. Indicate the average number of flights of stairs **climbed up** in the past year. Walking down stairs should not be recorded.

<table>
<tr><th></th><th>Did you participate in this activity in the past year?
1 Yes; 2 No</th><th>How many months did you do this activity per year?</th><th>How many days on average did you do this activity per month or per week?
|_|__|Day/Mon / |
|day/Week</th><th>How long (H:M) on average did you do this activity each day?
|_||_|:|_|_|</th></tr>
<tr><td>1. At home</td><td>|___|</td><td>|___|___|</td><td>|___|___|/|___|</td><td>|___|___|___|___|</td></tr>
<tr><td>2. At working unit/ school</td><td>|___|</td><td>|___|___|</td><td>|___|___|/|___|</td><td>|___|___|___|___|</td></tr>
</table>

2. Are there any other activities that you do often that were NOT included in the above questions? If yes, please fill in the next table.

<table>
<tr><th></th><th>Did you participate in this activity in the past year?
1 Yes; 2 No</th><th>How many months did you do this activity per year?</th><th>How many days on average did you do this activity per month or per week?
|_|__|Day/Mon / |
|day/Week</th><th>How long (H:M) on average did you do this activity each day?
|_||_|:|_|_|</th></tr>
<tr><td>Activity 1</td><td>|__|</td><td>|__|__|</td><td>|__|__|/|__|</td><td>|__|__|:|__|__|</td></tr>
<tr><td>Activity 2</td><td>|__|</td><td>|__|__|</td><td>|__|__|/|__|</td><td>|__|__|:|__|__|</td></tr>
<tr><td>Activity 3</td><td>|__|</td><td>|__|__|</td><td>|__|__|/|__|</td><td>|__|__|:|__|__|</td></tr>
</table>

3. Did you have difficulty understanding any of the questions we asked?
   [1] Yes [2] No
   If yes, please indicate the questions and the reason(s) for difficulty ____.

4. Did you have difficulty answering any of the questions we asked? For example, was there anything we asked that you had to think about a lot, or that was hard for you to remember or estimate the answer to the question?
   [1] Yes [2] No
   If yes, record the question and the reason(s) you found the question difficult to answer : ____.

5. Was there anything about the choices for response that you found unclear?
   [1] Yes [2] No
   If yes, record the answer and the reason(s) you found the choices for response unclear to understand:____.

6. Was there anything else that you think we should have done differently for this study, including the way we contacted you, the way the study was explained to you or the interview?

[1] Yes [2] No

If yes, please indicate what could have been done differently.____.

Ending time:__(H):__(M) |__|__|__|__|	Time to be used:__(H)__(M) |__|__|__|__|

Supplementary Table S2. China-US Collaboration Project on Chinese Children and Families Cohort Study Physical Activity Questionnaire (Chinese Version)

过去一年体力活动调查问卷

受试者编号：|__|__|__|__||__|__|__|__|

调查日期：___年_月_日 |__|__|__|__|__|__|__|__| 调查员：__ |__|__|__|

在下面几页中我们将询问您过去一年（自今天起倒推12个月内）的体力活动情况，包括家务、工作、学校、休闲、锻炼等方面。如果参加了某项活动，在对应问题下圈"是"，并记录进行此项活动的频率与时间。对没有参加的活动，圈"否"并跳到下一问题。

A.居家活动（我们会在问卷的后面询问在学校的进行体力活动的情况）：

1.坐着：看电视、录像或影碟<br>1…不是 2…是 |__|	一年中有几个月进行该活动？<br>___月 |__|__|	进行该活动的月份中，平均每月（或每周）有几天进行该活动？<br>___天/月 |__|__| 或者___天/周 |__|__|	这些天中平均每天进行多长时间？<br>___：___ |__|__|__|__|

2.坐着：看书、织缝、与家人或朋友闲聊（非上班或者在学校）<br>1…不是　2…是　|__|	一年中有几个月进行该活动？<br>___月　|__|__|	进行该活动的月份中，平均每月（或每周）有几天进行该活动？<br>___天/月 |__|__| 或者___天/周　|__|__|	这些天中平均每天进行多长时间？<br>___ ：___ |__|__|__|__|

2b.打电脑（非上班或者在学校）<br>1…不是　2…是　|__|	一年中有几个月进行该活动？<br>___月　|__|__|	进行该活动的月份中，平均每月（或每周）有几天进行该活动？<br>___天/月 |__|__| 或者___天/周　|__|__|	这些天中平均每天进行多长时间？<br>___ ：___ |__|__|__|__|

3.简单的日常琐事：做饭、打扫、除尘、洗衣、外出购物（仅指在商场中的时间，不包括来去路上的时间）、养花草、简单地照顾动物。<br>1…不是 2…是 |__|	一年中有几个月进行该活动？<br>___月 |__|__|	进行该活动的月份中，平均每月（或每周）有几天进行该活动？<br>___天/月 |__|__| 或者___天/周 |__|__|	这些天中平均每天进行多长时间？<br>___：___ |__|__|__|__|

4.中、重度活动：大扫除（擦地板、擦窗、吸尘、拖地）、洗车、农活，照顾动物等。<br>1…不是 2…是 |__|	一年中有几个月进行该活动？<br>___月 |__|__|	进行该活动的月份中，平均每月（或每周）有几天进行该活动？<br>___天/月 |__|__| 或者___天/周 |__|__|	这些天中平均每天进行多长时间？<br>___：___ |__|__|__|__|

B.步行：

1.步行上下班<br>1…不是 2…是 |__|	一年中有几个月进行该活动？<br>___月 |__|__|	进行该活动的月份中，平均每月（或每周）有几天进行该活动？<br>___天/月 |__|__| 或者___天/周 |__|__|	这些天中平均每天进行多长时间？<br>___：___ |__|__|__|__|

1b.步行上学和放学回家<br>1…不是 2…是 |__|	一年中有几个月进行该活动？<br>___月 |__|__|	进行该活动的月份中，平均每月（或每周）有几天进行该活动？<br>___天/月 |__|__| 或者___天/周 |__|__|	这些天中平均每天进行多长时间？<br>___：___ |__|__|__|__|

2.步行外出（购物/办事）<br>1…不是　2…是　|__|	一年中有几个月进行该活动？<br>___月　|__|__|	进行该活动的月份中，平均每月（或每周）有几天进行该活动？<br>___天/月 |__|__| 或者___天/周　|__|__|	这些天中平均每天进行多长时间？<br>___：___ |__|__|__|__|

3.以锻炼为目的的散步和慢跑步行<br>1…不是　2…是　|__|	一年中有几个月进行该活动？<br>___月　|__|__|	进行该活动的月份中，平均每月（或每周）有几天进行该活动？<br>___天/月 |__|__| 或者___天/周　|__|__|	这些天中平均每天进行多长时间？<br>___：___ |__|__|__|__|

C.出行活动（指上班、上学、日常外出以及旅游）

1.骑自行车<br>1…不是 2…是 |__|	一年中有几个月进行该活动？<br>___月 |__|__|	进行该活动的月份中，平均每月（或每周）有几天进行该活动？<br>___天/月 |__|__| 或者___天/周 |__|__|	这些天中平均每天进行多长时间？<br>___：___ |__|__|__|__|

2.骑摩托车/助动车/电动车<br>1…不是 2…是 |__|	一年中有几个月进行该活动？<br>___月 |__|__|	进行该活动的月份中，平均每月（或每周）有几天进行该活动？<br>___天/月 |__|__| 或者___天/周 |__|__|	这些天中平均每天进行多长时间？<br>___：___ |__|__|__|__|
3.乘公交、地铁、或开车<br>1…不是 2…是 |__|	一年中有几个月进行该活动？<br>___月 |__|__|	进行该活动的月份中，平均每月（或每周）有几天进行该活动？<br>___天/月 |__|__| 或者___天/周 |__|__|	这些天中平均每天进行多长时间？<br>___：___ |__|__|__|__|

D.工作相关的活动（包括义务工作）（请跳过这个问题，如果你没有工作或者没有从事志愿者工作，我们将在后面的部分询问上学情况）

1.坐着：看书、写字、打电脑、伏案工作、开会、做实验等<br>1…不是 2…是 |__|	一年中有几个月进行该活动？<br>___月 |__|__|	进行该活动的月份中，平均每月（或每周）有几天进行该活动？<br>___天/月 |__|__| 或者___天/周 |__|__|	这些天中平均每天进行多长时间？<br>___：___ |__|__|__|__|

2.缓慢/间断的行走（指站立或连续步行少于 5 分钟。例如营业员、复印文件、办公室内走动、简单的园艺活动和农活，简单地照顾动物等<br>1…不是 2…是 |__|	一年中有几个月进行该活动？<br>___月 |__|__|	进行该活动的月份中，平均每月（或每周）有几天进行该活动？<br>___天/月 |__|__| 或者___天/周 |__|__|	这些天中平均每天进行多长时间？<br>___ ：___ |__|__|__|__|

3.站立—轻度或中度体力劳动：端盘子、健康服务、清洁工、推拿按摩、其他搬运小于 50 斤的物品的活动。园艺活动，务农，钓鱼，照顾动物<br>1…不是 2…是 |__|	一年中有几个月进行该活动？<br>___月 |__|__|	进行该活动的月份中，平均每月（或每周）有几天进行该活动？<br>___天/月 |__|__| 或者___天/周 |__|__|	这些天中平均每天进行多长时间？<br>___ ：___ |__|__|__|__|

4.站立—中度/重度体力劳动：搬运、装卸搬运大于 50 斤物品的活动，一般建筑工人、卡车司机、搬运工从事的工作。繁重的农活和园艺活动<br>1…不是 2…是 |__|	一年中有几个月进行该活动？<br>___月 |__|__|	进行该活动的月份中，平均每月（或每周）有几天进行该活动？<br>___天/月 |__|__| 或者___天/周 |__|__|	这些天中平均每天进行多长时间？<br>___ ：___ |__|__|__|__|

E.照顾老人及小孩；饲养宠物

1.轻体力活动：洗澡、喂饭、陪伴玩耍等<br>1…不是　2…是　|__|	一年中有几个月进行该活动？<br>___月　|__|__|	进行该活动的月份中，平均每月（或每周）有几天进行该活动？<br>___天/月 |__|__| 或者___天/周　|__|__|	这些天中平均每天进行多长时间？<br>___ ：___　|__|__|__|__|

2.中度体力活动：抬、背、推轮椅/婴儿车、遛狗、遛鸟等<br>1…不是　2…是　|__|	一年中有几个月进行该活动？<br>___月　|__|__|	进行该活动的月份中，平均每月（或每周）有几天进行该活动？<br>___天/月 |__|__| 或者___天/周　|__|__|	这些天中平均每天进行多长时间？<br>___ ：___　|__|__|__|__|

3.中度至重度的活动：照顾动物，比如清洗笼子、赶羊、赶牛、赶猪、赶鸭子以及养殖活动等<br>1…不是　2…是　|__|	一年中有几个月进行该活动？<br>___月　|__|__|	进行该活动的月份中，平均每月（或每周）有几天进行该活动？<br>___天/月 |__|__| 或者___天/周　|__|__|	这些天中平均每天进行多长时间？<br>___ ：___　|__|__|__|__|

如果你现在正在上学，或者过去一年在学校，请跳至问题 G 回答。

F.业余活动、休闲活动、体育锻炼（工作之外）

1.轻缓型锻炼：慢跑、太极拳、木兰拳、剑舞、扇舞等<br>1…不是　2…是　|__|	一年中有几个月进行该活动？<br>___月　|__|__|	进行该活动的月份中，平均每月（或每周）有几天进行该活动？<br>___天/月 |__|__| 或者___天/周　|__|__|	这些天中平均每天进行多长时间？<br>___ ：___　|__|__|__|__|

2.跳舞（以休闲或社交为目的）<br>1…不是　2…是　|__|	一年中有几个月进行该活动？<br>___月　|__|__|	进行该活动的月份中，平均每月（或每周）有几天进行该活动？<br>___天/月 |__|__| 或者___天/周　|__|__|	这些天中平均每天进行多长时间？<br>___ ：___　|__|__|__|__|
3.中等强度球类运动：保龄球、桌球、乒乓球等<br>1…不是　2…是　|__|	一年中有几个月进行该活动？<br>___月　|__|__|	进行该活动的月份中，平均每月（或每周）有几天进行该活动？<br>___天/月 |__|__| 或者___天/周　|__|__|	这些天中平均每天进行多长时间？<br>___ ：___　|__|__|__|__|

4.高强度球类运动：篮球、足球、羽毛球、网球等<br>1…不是　2…是　|__|	一年中有几个月进行该活动？<br>___月　|__|__|	进行该活动的月份中，平均每月（或每周）有几天进行该活动？<br>___天/月 |__|__| 或者___天/周　|__|__|	这些天中平均每天进行多长时间？<br>___：___　|__|__|__|__|

5.中等强度有氧锻炼：低强度跳操、慢速游泳、简单的踏车、走跑器械等　1…不是　2…是　|__|	一年中有几个月进行该活动？<br>___月　|__|__|	进行该活动的月份中，平均每月（或每周）有几天进行该活动？<br>___天/月 |__|__| 或者___天/周　|__|__|	这些天中平均每天进行多长时间？<br>___：___　|__|__|__|__|

6.高强度有氧锻炼：高强度跳操、快速持久的游泳、长跑等　1…不是　2…是　|__|	一年中有几个月进行该活动？<br>___月　|__|__|	进行该活动的月份中，平均每月（或每周）有几天进行该活动？<br>___天/月 |__|__| 或者___天/周　|__|__|	这些天中平均每天进行多长时间？<br>___：___　|__|__|__|__|

7.力量型锻炼：俯卧撑、举重、哑铃、拉力器等<br>1…不是　2…是　|__|	一年中有几个月进行该活动？<br>___月　|__|__|	进行该活动的月份中，平均每月（或每周）有几天进行该活动？<br>___天/月 |__|__| 或者___天/周　|__|__|	这些天中平均每天进行多长时间？<br>___：___　|__|__|__|__|

如果你已不上学，跳至问题 I 回答。

如果你现在正在上学，或者过去一年在学校，请回答以下内容

G.学校活动

1.坐着：听课、看书、写字、打电脑、做实验等其他伏案活动<br>1…不是　2…是　|__|	一年中有几个月进行该活动？<br>___月　|__|__|	进行该活动的月份中，平均每月（或每周）有几天进行该活动？<br>___天/月 |__|__| 或者___天/周　|__|	这些天中平均每天进行多长时间？<br>___：___　|__|__|__|__|

2.站在或缓慢步行：实验室工作、在教室来回走动等<br>1…不是　2…是　|__|	一年中有几个月进行该活动？<br>___月　|__|__|	进行该活动的月份中，平均每月（或每周）有几天进行该活动？<br>___天/月 |__|__| 或者___天/周　|__|__||	这些天中平均每天进行多长时间？<br>___：___　|__|__|__|__|
3.从一个教室走到另一个教室：打扫卫生，户外学习活动等<br>1…不是　2…是　|__|	一年中有几个月进行该活动？<br>___月　|__|__|	进行该活动的月份中，平均每月（或每周）有几天进行该活动？<br>___天/月 |__|__| 或者___天/周　|__|__|	这些天中平均每天进行多长时间？<br>___：___　|__|__|__|__|

4.高强度活动：园艺活动、打扫教室、照顾动物、做饭等<br>1…不是　2…是　|__|	一年中有几个月进行该活动？<br>___月　|__|__|	进行该活动的月份中，平均每月（或每周）有几天进行该活动？<br>___天/月 |__|__| 或者___天/周　|__|__|	这些天中平均每天进行多长时间？<br>___：___　|__|__|__|__|

H.业余活动、休闲活动、体育锻炼（校外）

1.轻缓型锻炼：慢跑、太极拳、木兰拳、剑舞、扇舞等<br>1…不是 2…是 |_|	一年中有几个月进行该活动？<br>___月 |_|_|	进行该活动的月份中，平均每月（或每周）有几天进行该活动？<br>___天/月 |_|_| 或者___天/周 |_|_|	这些天中平均每天进行多长时间？<br>___：___ |_|_|_|_|

2.跳舞（以休闲或社交为目的）<br>1…不是 2…是 |_|	一年中有几个月进行该活动？<br>___月 |_|_|	进行该活动的月份中，平均每月（或每周）有几天进行该活动？<br>___天/月 |_|_| 或者___天/周 |_|_|	这些天中平均每天进行多长时间？<br>___：___ |_|_|_|_|
3.中等强度球类运动：保龄球、桌球、乒乓球等<br>1…不是 2…是 |_|	一年中有几个月进行该活动？<br>___月 |_|_|	进行该活动的月份中，平均每月（或每周）有几天进行该活动？<br>___天/月 |_|_| 或者___天/周 |_|_|	这些天中平均每天进行多长时间？<br>___：___ |_|_|_|_|

4.高强度球类运动：篮球、足球、羽毛球、网球等<br>1…不是 2…是 |_|	一年中有几个月进行该活动？<br>___月 |_|_|	进行该活动的月份中，平均每月（或每周）有几天进行该活动？<br>___天/月 |_|_| 或者___天/周 |_|_|	这些天中平均每天进行多长时间？<br>___：___ |_|_|_|_|

5.中等强度有氧锻炼：低强度跳操、慢速游泳、简单的踏车、走跑器械等	一年中有几个月进行该活动？<br>___月 |_|_|	进行该活动的月份中，平均每月（或每周）有几天进行该活动？<br>___天/月 |_|_| 或者___天/周 |_|_|	这些天中平均每天进行多长时间？<br>___：___ |_|_|_|_|

1…不是　2…是　|__|			

6.高强度有氧锻炼：高强度跳操、快速持久的游泳、长跑等　1…不是　2…是　|__|	一年中有几个月进行该活动？<br>___月　|__|__|	进行该活动的月份中，平均每月（或每周）有几天进行该活动？<br>___天/月 |__|__| 或者___天/周　|__|__|	这些天中平均每天进行多长时间？<br>___：___　|__|__|__|__|

7.力量型锻炼：俯卧撑、举重、哑铃、拉力器等<br>1…不是　2…是　|__|	一年中有几个月进行该活动？<br>___月　|__|__|	进行该活动的月份中，平均每月（或每周）有几天进行该活动？<br>___天/月 |__|__| 或者___天/周　|__|__|	这些天中平均每天进行多长时间？<br>___：___　|__|__|__|__|

3.以锻炼为目的散步<br>1…不是　2…是　|__|	一年中有几个月进行该活动？<br>___月　|__|__|	进行该活动的月份中，平均每月（或每周）有几天进行该活动？<br>___天/月 |__|__| 或者___天/周　|__|__|	这些天中平均每天进行多长时间？<br>___：___　|__|__|__|__|

I.其他问题

1.您平均每天登多少级楼梯（仅计算登楼梯数，不包括下楼梯数）：

在家：___层___遍；
在单位：___层___遍；
在其他地方：___层___遍；
2.是否有你进行的其他体力活动没有涵盖在这份问卷中？如果有请填入下表：

体力活动	一年中有几个月进行该活动？<br>___月 |__|__|	进行该活动的月份中，平均每月（或每周）有几天进行该活动？<br>___天/月 |__|__| 或者___天/周 |__|__|	这些天中平均每天进行多长时间？<br>___：___ |__|__|__|__|
体力活动	一年中有几个月进行该活动？<br>___月 |__|__|	进行该活动的月份中，平均每月（或每周）有几天进行该活动？<br>___天/月 |__|__| 或者___天/周 |__|__|	这些天中平均每天进行多长时间？<br>___：___ |__|__|__|__|
体力活动	一年中有几个月进行该活动？<br>___月 |__|__|	进行该活动的月份中，平均每月（或每周）有几天进行该活动？<br>___天/月 |__|__| 或者___天/周 |__|__|	这些天中平均每天进行多长时间？<br>___：___ |__|__|__|__|
体力活动	一年中有几个月进行该活动？f72<br>___月 |__|__|	进行该活动的月份中，平均每月（或每周）有几天进行该活动？<br>f73	这些天中平均每天进行多长时间？<br>___：___ |__|__|__|__|

		___天/月 |__|__| 或者___天/周 |__|__|	

如果有需要，可以增加表格数目。

Supplementary Table S3. MET Scores for the Activities in the Physical Activity Questionnaire (PAQ).

Domain	Activities from the PAQ	Code	MET Score	Intensity
Household	Sitting/Lying: Watching TV or VCR/ VCD/ DVD	11	1.15	Sedentary
Household	Sitting: using computer (not at work), playing video games	12	1.25	Sedentary
Household	Sitting: eating, reading. knitting, sewing, chatting with family or friends, playing cards, chess or Majiang	13	1.49	Sedentary
Household	Light chores: Cooking, washing dishes, cleaning up, laundry, dusting, shopping (time in store, not including time spent on the way), light gardening	14	2.21	Light
Household	Moderate to vigorous effort: Heavily cleaning up rooms (brushing floor, cleaning windows, vacuuming, and washing floor, cleaning yard), washing a car	15	3.72	Moderate to vigorous
Household	Sleeping	16	0.95	Sleeping
Walking	Walking to and from work (including walking to and from bus station)	21	3.25	Moderate to vigorous
Walking	Out (Shopping/Chores): walking to and from household chores, including shopping, visiting friends	22	2.25	Light
Walking	Walking during leisure time (not at work, not walking dog or birds)	23	3.5	Moderate to vigorous
Transportation	Riding bike	31	6.8	Moderate to vigorous
Transportation	Driving motorcycle/scooter, car	32	2.65	Light
Transportation	Riding in a motorcycle/scooter, car, taxi, bus, subway	33	1.3	Sedentary
Caregiving	Light effort: bathing, feeding, playing with children (reading, drawing, toys)	41	2.36	Light
Caregiving	Moderate effort: Lifting and carrying, pushing wheelchair or stroller, playing children game (ball)	42	3.7	Moderate to vigorous
Caregiving	Caring for pets: walking dogs, bird and cleaning pets	43	3.2	Moderate to vigorous
Leisure	Tutorial class out school on language, math, English, drawing, chess	51	1.58	Sedentary
Leisure	Tutorial class out school on musical instrument	52	2.0	Light

Leisure	Mind/Body exercises with light effort: Tai Chi, Mulan, sword dancing, fan dancing	53	2.25	Light
Leisure	Dancing (including Tutorial class out school) social or folk dancing	54	5.16	Moderate to vigorous
Leisure	Sports- moderate effort: balling, table tennis	55	4.28	Moderate to vigorous
Leisure	Sports- vigorous effort: basketball, soccer, badminton, tennis	56	7.91	Moderate to vigorous
Leisure	Conditioning exercises- moderate effort: low impact aerobics, slow swimming, roller skating	57	4.43	Moderate to vigorous
Leisure	Conditioning exercises- vigorous effort: step aerobics, high intensity and long time lasting swimming, jogging , climbing hills, and rope jumping	58	8.39	Moderate to vigorous
Leisure	Strengthening exercises: lifting body, lifting weights, strength training	59	4.43	Moderate to vigorous
Farming	Sitting: knitting	61	2.03	Sedentary
Farming	Watering crops, herding animals	62	2.1	Light
Farming	Standing –Moderate farming: like weeding, spraying insecticide, trimming, sowing, fertilizing, spading, digging; feeding animals, fishing,	63	4.28	Moderate to vigorous
Farming	Standing: hard farming, like manual harvesting, chopping , cleaning poultry house and barn	64	7.05	Moderate to vigorous
School	Sitting: listening, reading, writing, drawing, using computer, hand work, sitting in the lab	71	1.5	Sedentary
School	Slow / intermittent walking (lasting less than 5 minutes at a time) in the classroom or standing with light activity, like chatting with students	72	1.95	Light
School	Standing: walking from classroom to classroom, cleaning classroom or schoolyard	73	3.27	Light
School	Sports: Light effort, like radio calisthenics, warming up and cooling down activites	74	2.55	Light to Moderate
School	Sports: moderate effort, like Wushu, ping-pong, kicking shuttle cock, dancing, volleyball	75	4.18	Moderate to vigorous
School	Sports: hard effort, like long-distance run, shuttle run rope jumping, basketball, football, badminton, high intensity aerobics,	76	8.23	Moderate to vigorous
School	Strengthening exercises: Push-up, sit-up, pull-up, throwing	77	4.21	Moderate

Occupational	Sitting work: reading, writing, desk/computer work, sitting in meetings and labs, knitting	81	1.41	Sedentary
Occupational	Slow / intermittent walking (lasting less than 5 minutes at a time, or standing very light work, like a store clerk, Xeroxing, filing,	82	2.5	Light
Occupational	Standing –Moderate work: like waitress, healthcare, cleaning and massaging and others carrying items weighing less than 25 kg.	83	3.76	Moderate to vigorous
Occupational	Standing – hard work: like lifting, carrying, or unloading items weighting greater than 25 kgs, often done by construction workers, truck drivers, movers, delivery persons; mining.	84	5.63	Moderate to vigorous
